# An Assessment of Three Different *In Situ* Oxygen Sensors for Monitoring Silage Production and Storage

**DOI:** 10.3390/s16010091

**Published:** 2016-01-14

**Authors:** Guilin Shan, Yurui Sun, Menghua Li, Kerstin H. Jungbluth, Christian Maack, Wolfgang Buescher, Kai-Benjamin Schütt, Peter Boeker, Peter Schulze Lammers, Haiyang Zhou, Qiang Cheng, Daokun Ma

**Affiliations:** 1 College of Information and Electrical Engineering, China Agricultural University, Key Lab of Agricultural Information Acquisition Technology, Ministry of Agriculture, 100083 Beijing, China; sgl@cau.edu.cn (G.S.); naaiocean@163.com (M.L.); zhouhy@cau.edu.cn (H.Z.); chengqiang@cau.edu.cn (Q.C.); madaokun@cau.edu.cn (D.M.); 2 Department of Agricultural Engineering, The University of Bonn, 53115 Bonn, Germany; kjungblu@uni-bonn.de (K.H.J.); c.maack@uni-bonn.de (C.M.); buescher@uni-bonn.de (W.B.); kschuet1@uni-bonn.de (K.-B.S.); boeker@uni-bonn.de (P.B.); lammers@uni-bonn.de (P.S.L.)

**Keywords:** oxygen (O_2_), carbon dioxide (CO_2_), galvanic oxygen cell (GOC), Clark oxygen electrodes (COE), Dräger chip measurement system (DCMS), silage

## Abstract

Oxygen (O_2_) concentration inside the substrate is an important measurement for silage-research and-practical management. In the laboratory gas chromatography is commonly employed for O_2_ measurement. Among sensor-based techniques, accurate and reliable *in situ* measurement is rare because of high levels of carbon dioxide (CO_2_) generated by the introduction of O_2_ in the silage. The presented study focused on assessing three types of commercial O_2_ sensors, including Clark oxygen electrodes (COE), galvanic oxygen cell (GOC) sensors and the Dräger chip measurement system (DCMS). Laboratory cross calibration of O_2_ *versus* CO_2_ (each 0–15 vol.%) was made for the COE and the GOC sensors. All calibration results verified that O_2_ measurements for both sensors were insensitive to CO_2_. For the O_2_ *in situ* measurement in silage, all O_2_ sensors were first tested in two sealed barrels (diameter 35.7 cm; height: 60 cm) to monitor the O_2_ depletion with respect to the ensiling process (Test-A). The second test (Test-B) simulated the silage unloading process by recording the O_2_ penetration dynamics in three additional barrels, two covered by dry ice (0.6 kg or 1.2 kg of each) on the top surface and one without. Based on a general comparison of the experimental data, we conclude that each of these *in situ* sensor monitoring techniques for O_2_ concentration in silage exhibit individual advantages and limitations.

## 1. Introduction

Silage, mainly made from maize or grass or alfalfa, is a preferred food for dairy cattle and also provides supplemental forage or roughage for beef cattle. Silage is regarded as an economical feedstuff because it contains plenty of protein and high energy (mostly starch) in terms of cost per unit of protein or energy [1,2]. On the other hand, silage is very sensitive to oxygen (O_2_) intrusion because it contains aerobic bacteria, yeasts and moulds under anaerobic conditions. When the silo opens for feeding, silage is exposed to air, and fermentation acids and other substrates are oxidized quickly by the aerobic microorganisms, resulting first in dry matter and nutrient losses, followed by generation of mould spores and toxins [1,2,3,4]. Silage aerobic stability is of critical importance to maintain feedstuff safety and cattle health [1,2].

To monitor aerobic deterioration during silage production, the internal temperature of silage is often used as an indicator or alarm. Abnormal temperature rises result from heat released by microbial activity inside the silage as entrained O_2_ is consumed by microbial respiration [5,6,7,8]. According to previous research [9], there is a significant lag (hours long) between the peak O_2_ concentration and the maximum temperature rise. Moreover, the temperature rise could be affected by the silage water content, pH-value or buffering capacity of silage [10]. As a consequence, serious silage spoilage may occur before the silage temperature reaches the maximum value. Therefore, relying on the O_2_–induced silage temperature rise as an alarm for aerobic deterioration, is of limited value in reducing dry matter and nutrient losses from silage, whereas O_2_ or CO_2_ concentration may be the most sensitive, timely and effective indictors [9].

Regarding O_2_ measurement in silage, a common method is to extract gas samples from the silage which are later analyzed using a laboratory gas chromatograph [3,11,12,13]. Even though the gas chromatography method is accurate, two technical issues should be considered. First, sample extraction may result in additional O_2_ entering the silage. Second, the method is time consuming and thus unable to track silage O_2_ dynamics. A recent study proposed the Clark oxygen electrodes (COE) for continuous measurement of O_2_ concentration [9]. Their preliminary experimental results indicated that the COE could be used not only for dissolved O_2_ concentration in liquids, but was also found promising for measuring the gaseous O_2_ concentration in silage. The latter is equally important but has received less attention in wide applications of the COE. A major advantage of the COE is the ability to electronically track O_2_ dynamics in the silage. However, unlike O_2_ measurements in the atmosphere where CO_2_ concentration is usually negligible (≤0.1 vol.%), high levels of CO_2_ concentration in silage are often produced by microorganisms [14,15]. Thus, an important work in concluding that a sensor is capable of measuring O_2_ in silage, would be a cross-calibration of O_2_ concentration relative to CO_2_. However, such a study is lacking in the literature [9]. In addition, nowadays diverse types of commercial O_2_ sensors for atmospheric measurement have been made available [16,17,18,19]. Besides the COE, other existing O_2_ sensors could also be promising or even better for monitoring O_2_ concentration in silage. Based on these considerations, as a continuation of previous research with the same intention, our research objectives were to evaluate three types of O_2_ sensors, including galvanic cell oxygen (GOC) sensors and a Dräger chip measurement system (DCMS) in addition to the COE.

## 2. Materials and Methods

### 2.1. Brief Description of the Tested O_2_ Sensors

**Figure 1:**
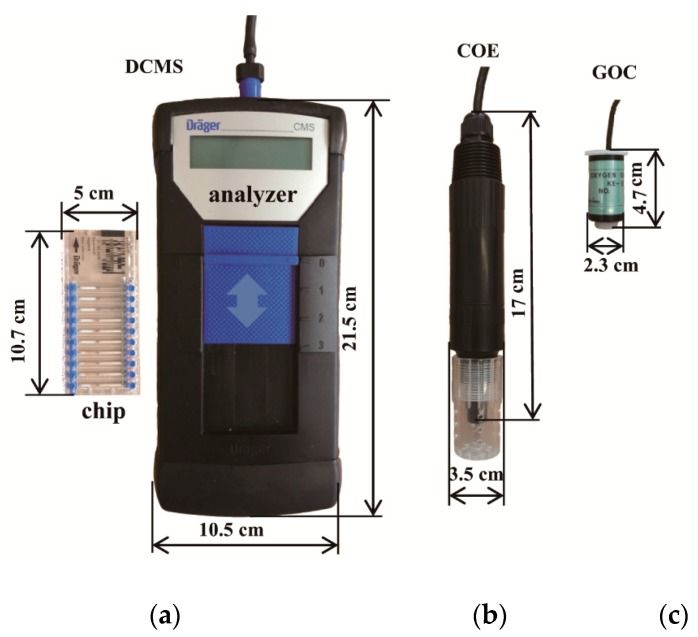
Photos and dimensions of the three types of oxygen (O_2_) sensors, (**a**) Dräger chip measurement system (DCMS); (**b**) the Clark oxygen electrodes(COE); (**c**) galvanic oxygen cell (GOC).

The photos in Figure 1 show three O_2_ sensors tested in this study. The COE consists of a sensing platinum electrode (cathode) and a reference silver electrode (anode), which are immersed in saturated KCl electrolyte and covered with a gas-permeable membrane [16,20]. According to the reactions listed in Table 1, when the O_2_ diffuses through the gas permeable membrane to the cathode, it is reduced to hydroxide ions resulting in an amperometric signal (fractions of 10^2^ nA) that has a linear relationship with the O_2_ concentration. The signal-conditioning unit of the COE contains a microampere amplifier and a current-to-voltage converter, which outputs a voltage signal (fractions of 1 V) for users.

**Table 1:** General information and specifications of the three types of O_2_ sensors.

Sensor	Type	Manufacturer	Output	Accuracy	Reaction	Electrolyte
COE	DO-10	UNISM Technologies Inc., Beijing, China	100–800 (mV)	±0.2 mg/L	4Ag + 4Cl^−^ → 4AgCl + 4e^−^ (anode) O_2_ + 2H_2_O + 4e^−^ → 4OH^−^ (cathode)	KCl
GOC	KE-50	FIGARO Engineering Inc., Osaka, Japan	0–65.0 (mV)	±2 vol.% of full scale	2Pb + 2H_2_O → 2PbO + 4H^+^ + 4e^−^ (anode) O_2_ + 4H^+^ + 4e^−^ → 2H_2_O (cathode)	H_2_SO_4_
DCMS	6406490	Dräger Safety AG & Co. KGaA., Lübeck, Germany	1–30 vol.%	±15 vol.% of the measured value	O_2_ + TiCl_3_ → Ti^IV^compound + HCl	None

The COE is commonly designed for measuring dissolved oxygen concentration (DOC) in liquids. As DOC is a function of temperature of the liquid measured, a temperature sensor is commonly integrated in the COE. Therefore, all commercial COE sensors can provide paired signals to simultaneously track the dynamics of O_2_ concentration and temperature (T_si_) in silage without additional cost. As a matter of fact, the membrane is O_2_-permeable only by gases, essentially the COE measurement relates to the gaseous O_2_. The DOC (mg·L^−1^) in solution is directly proportional to the partial pressure of O_2_ in the air (p, kPa) based on the Henry’s law of physical chemistry [9].

With a similar structure to the COE but an alternative principle of sensing O_2_, the GOC also has an anode (oxidation resulting in loss of electrons) and a cathode (reduction resulting in gain of electrons), both electrodes immersed in a specific electrolyte. When O_2_ diffuses into the sensor, a quantitative reduction of O_2_ occurs at the cathode (reaction listed in Table 1). As a result, the electrons required for the O_2_ reduction flow through the external circuit from the anode, where an equal magnitude oxidation reaction takes place. The resulting electric current yields an output signal. The potential of the cathode is established by the use of an anode material, such as lead or cadmium which is sufficiently electronegative in the electrochemical series [21]. Based on its measurement principle, the GOC is electrically equivalent to a primary battery or a fuel cell whose output voltage is linearly proportional to the O_2_ concentration or activity at the electrode [16,17,18,19]. Different from the COE designed for the DOC measurement, the GOCs are mainly designed for gaseous O_2_ measurements in the atmosphere so the temperature influence/correction is not considered in its design [16,18].

**Figure 2:**
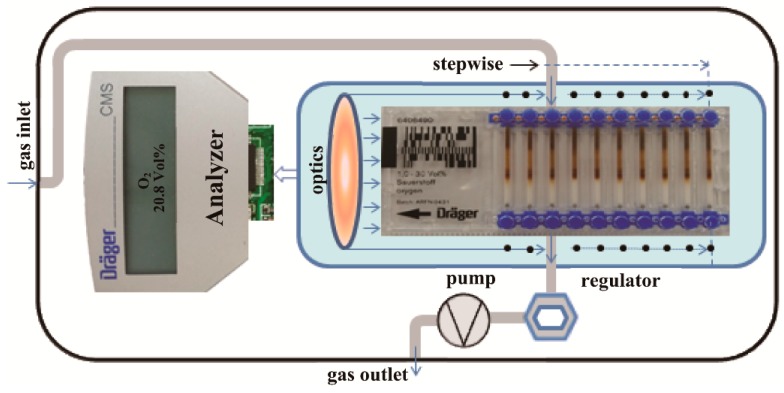
Schematic illustration of the physical structure, measurement principle and operating process of the DCMS.

Figure 2 illustrates the measurement principle and operating process of DCMS, which consists of a series of substance-specific measuring chips, and an electronic based analyzer [22]. Each type of chip targets a specific gas and contains 10 capillaries (*i.e.*, for 10 measurements) filled with a chemical reagent based on a colorimetric principle and proprietary algorithms. Table 1 lists the chemical reaction of the O_2_ chip used.

The analyzer combines an advanced optical system for identifying the color reaction by means of a mass flow controller and a special pump system. When a chip is inserted into the instrument, first the analyzer automatically determines the measurement gas target through a bar code attached on the chip. As a measurement starts, the pump system pulls the gas at a constant mass-flow (15 mL·min^−1^) passing the capillary through an air-tight connection between the entire gas conduction system and the open capillary of the chip. The optoelectronic detectors dynamically evaluate the reaction effects in the chip capillary, and eventually the result of the measurement appears on the displayer. A measurement needs a period of 2~4 min, depending on the actual O_2_ concentration. For a measurement with two minutes, 30 mL of gas must be sampled. The reading is the volumetric concentration of O_2_ so that specific calibration is not required [22].

### 2.2. Cross-Calibration System for O_2_ Versus CO_2_

**Figure 3:**
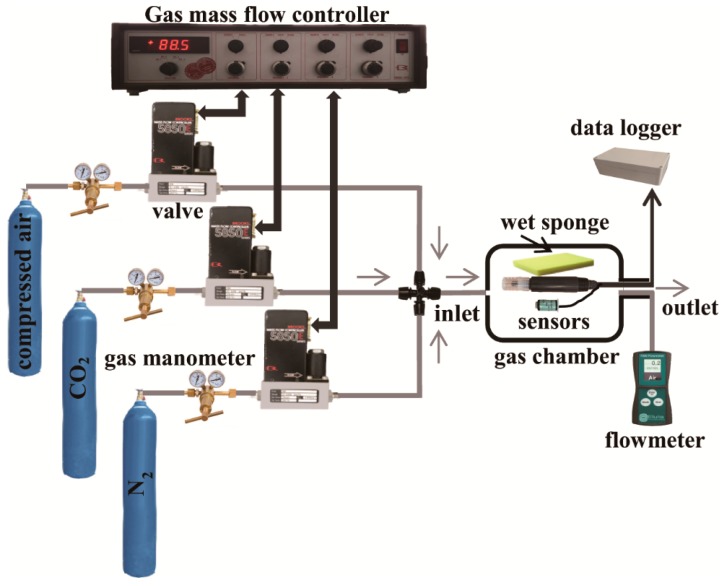
Setup of the cross calibration system used for the COE and the GOC. The mixture in gas chamber consists of O_2_, N_2_ and CO_2_ in volumetric ratio required.

Figure 3 illustrates the setup of the cross-calibration system, included a gas mass flow controller together with three gas valves (Model 5850E, Brooks Instrument, Chelmsford, MA, USA), a gas flow meter (7000 GC Flowmeter, Ellutia Chromatography Solutions, Cambs, UK) and a plastic gas chamber. 

Before starting calibration, the mass flow rate of each gas valve connecting to a compressed air, CO_2_ or N_2_ pipe, was predetermined according to the volumetric ratio of the gas mixture components. After adjusting each valve to a target concentration, the mixture in the gas chamber stabilized within ten minutes. In addition, considering that the air relative humidity in silage is rather high (70% to 95%) [11], a piece of sufficiently wet sponge (15 × 6 × 6 cm) was placed beside the calibrated sensors in the gas chamber. In this study, both the COE and the GOC were calibrated using this gas flow system. There are two reasons for the DCMS to be excluded from the calibration. First, the DCMS manual stated that the calibration is not required because its output directly displays the volumetric ratio (vol.%) of O_2_, so a calibration-based conversion between the sensor output and the display is unnecessary. Second, the DCMS has a gas intake demand which exceeds the flow capacity of the gas flowing/mixing system, and therefore this calibration system is not suitable for checking the DCMS accuracy.

### 2.3. Cross Calibration of O_2_ Versus CO_2_

For a gas mixed (*GM_i_*) with O_2_, N_2_ and CO_2_, *GM_i_* can be expressed as:

(1) $$GM_{i}=\begin{array}{ccc} (\alpha_{i}, & \beta_{i}, & \gamma_{i}) \end{array}\left(\begin{array}{c} O_{2}\\ N_{2}\\ {\text{CO}}_{2} \end{array}\right)$$

Suppose n gaseous samples (*GM*_Σ_) are prepared for the cross calibration of O_2_ *versus* CO_2_, *GM*_Σ_ can be presented as:

(2) $$GM_{\Sigma }=\left(\begin{array}{c} GM_{1}\\ GM_{2}\\ \vdots \\ GM_{n} \end{array}\right)=(\begin{array}{c} \alpha_{1}\\ \alpha_{2}\\ \vdots \\ \alpha_{n} \end{array}\begin{array}{c} \beta_{1}\\ \beta_{2}\\ \vdots \\ \beta_{n} \end{array}\begin{array}{c} \gamma_{1}\\ \gamma_{2}\\ \vdots \\ \gamma_{n} \end{array})\left(\begin{array}{c} O_{2}\\ N_{2}\\ {\text{CO}}_{2} \end{array}\right)$$

where α*_i_*, β*_i_* and γ*_i_* are the volume fractions of O_2_, N_2_ and CO_2_ in the mix, respectively, and a restriction follows:

(3) $$\begin{array}{r} \alpha_{i}+\beta_{i}+\gamma_{i}\approx 1\\ i=1,\;2,\cdots n \end{array}$$

In principle, the equations above fit the cross calibration under arbitrary volume fractions of O_2_, N_2_ and CO_2_. However, because of the flammable nature of pure O_2_, we used atmospheric air as the O_2_ source in the calibration, where atmospheric air is described by:

(4) $$air\approx \begin{array}{ccc} (0.2095, & 0.7808, & 0.0003) \end{array}\left(\begin{array}{c} O_{2}\\ N_{2}\\ {\text{CO}}_{2} \end{array}\right)$$

this substitution is similar to a mathematical base transform written as:

(5) $$GM_{\Sigma }=\left(\begin{array}{c} GM_{1}\\ GM_{2}\\ \vdots \\ GM_{n} \end{array}\right)=(\begin{array}{c} \alpha_{a1}\\ \alpha_{a2}\\ \vdots \\ \alpha_{an} \end{array}\begin{array}{c} \beta_{a1}\\ \beta_{a2}\\ \vdots \\ \beta_{an} \end{array}\begin{array}{c} \gamma_{a1}\\ \gamma_{a2}\\ \vdots \\ \gamma_{an} \end{array})\left(\begin{array}{c} \text{air}\\ N_{2}\\ {\text{CO}}_{2} \end{array}\right)$$

where α*_ai_*, β*_ai_* and γ*_ai_* also fulfill the restriction with:

(6) $$\begin{array}{c} \alpha_{ai}+\beta_{ai}+\gamma_{ai}\approx 1\\ \;i=1,\;2,\cdots n \end{array}$$

Because air is mainly made from N_2_ (78 vol.%) and O_2_ (20.95 vol.%), for the cross-calibration of O_2_ *versus* CO_2_, the gaseous samples (*GM*_Σ_) were prepared with combinations of O_2_ = 0, 5, 10, 15 vol.% and CO_2_ = 0, 5, 10, 15 vol.% (*i.e.*, *n* = 16), respectively. Prior to calibration, we measured ambient O_2_ using the DCMS and the average of four replications (O_2_ = 20. 88 vol.%) was used as the initial value in Equation (4).

### 2.4. Silage Material and Experimental Layout

The silage making process is generally divided into four phases: (i) the ensiling phase (a couple of hours) related to the O_2_ depletion process; (ii) the fermentation phase (about forty days) under anaerobic conditions; (iii) the stable and anaerobic storage phase (several months); and (iv) the feed-out phase/unloading phase/aerobic spoilage phase (a couple of days), which is related to the O_2_ penetrating process in silage [1,4]. Our experiment included two tests; Test-A, an analogue to the ensiling phase using fresh chopped maize material and Test-B, an analogue to the unloading phase using ensiled maize material.

**Figure 4:**
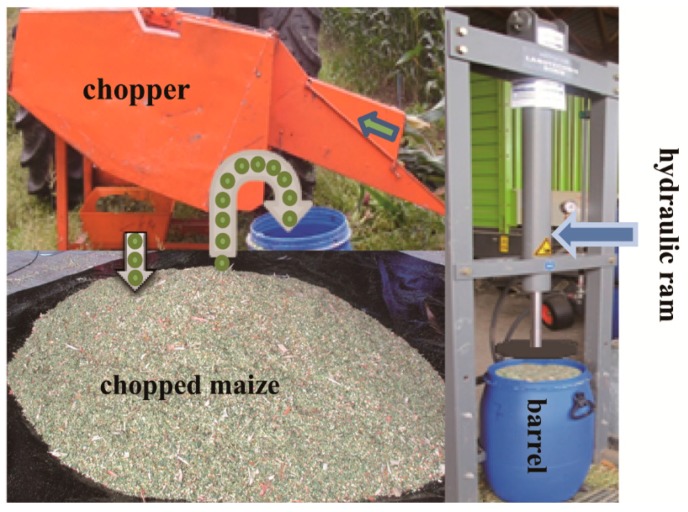
Setup of material preparation for Test-A with respect to ensiling process: Harvested maize was fed into a chopper. Then the chopped maize material was packed into a barrel using a hydraulic ram.

For Test-A, whole plants from freshly harvested maize were finely chopped using a PTO-driven chopper (Figure 4). The resulting biomass was packed into two polypropylene barrels (i.d.: 35.7 cm, length: 60 cm, vol. 60 L) using a hydraulic ram attached to a 34 cm diameter circular foot, in approximately six layer increments to ensure uniform density. One barrel was packed to a high wet bulk density (BD) (800 kg·m^−3^) and the other to a lower wet BD (500 kg·m^−3^). Three holes were drilled in the lid of each barrel using a hole-saw for the installation of the COE, the GOC and a rubber tube (o.d. 7 mm, i.d. 4 mm). The biomass in each barrel was also removed down to a depth of 10 cm, which allowed insertion of the COE and the GOC. In addition, one end of the rubber tube was connected to the DCMS and the other was inserted into each packed barrel at the same depth as the tip of COE and the GOC. All of the holes were sealed against the lid using sealant oil. After each barrel lid was closed, O_2_ trapped in the barrel was gradually consumed by the maize biomass until completely depleted.

For Test-B, maize silage was taken from 20 cm behind the silage face in a concrete bunker silo (40 × 6 × 3.5 m) at the research farm of Frankenforst (University of Bonn, Germany), being unloaded at a rate of approximately 0.5 m per day. The silage was re-ensiled in three additional 60 L barrels. These barrels were packed with high wet BDs (about 800 kg·m^−3^), then sealed and placed vertically on a bench. For installing the COE, the GOC and the rubber tube, three holes (depth: 10 cm) were drilled on the wall of each barrel 20-cm behind the silage face. After each barrel was sealed, O_2_ concentrations were monitored for 24 h until the internal O_2_ fully consumed. The lids of these barrels were then removed, the face of one barrel remained open while the other two were immediately covered with dry ice of 0.6 kg or 1.2 kg, respectively. Dry ice was used because it is a solid form of CO_2_ at very low temperature (−78.5 °C at earth atmospheric pressure) and completely sublimates unlike ice, providing a high concentration of CO_2_ at the interface. Therefore, dry ice placed at the boundary of the silage face acted as a partial barrier to O_2_ diffusion [5].

## 3. Results and Discussion

### 3.1. Results from the Cross-Calibration for the COE and the GOC

**Figure 5:**
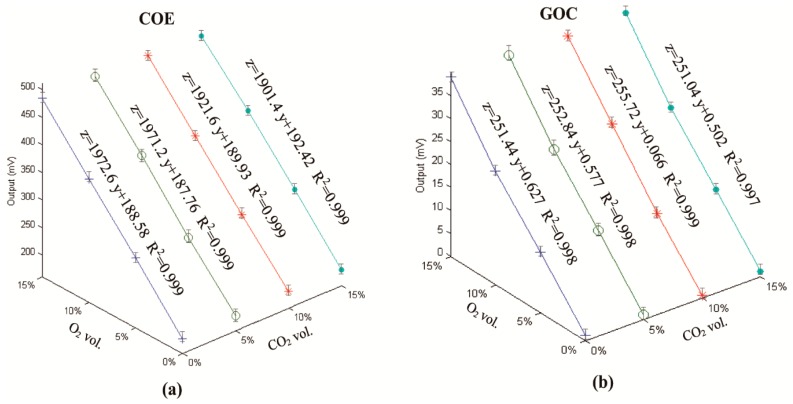
The cross calibration results with four levels of CO_2_ from the COE (**a**) and the GOC (**b**).

Figure 5a,b show the cross-calibration results with O_2_ *versus* CO_2_ for the COE and for the GOC, respectively. Three clear observations can be made. (i) Both sensors have good linear characteristics (for all linear equations R^2^ > 0.995). This confirmed that these electrochemical sensors can be calibrated using two points despite their use of different electrolytes [23,24,25,26], e.g., O_2_ = 0 vol.% in the pure N_2_ and O_2_ = 20.95 vol.% in air; (ii) Figure 5 assures that this type of COE is well-suited for measuring gaseous O_2_ as well as dissolved O_2_ [9]. This is especially significant when the COE is used in porous materials such as maize silage, rather than in liquids; (iii) The small variations in the slopes and intercepts of all calibration equations demonstrate that both types of O_2_ sensors are insensitive to CO_2_ concentrations ranging from 0 to 15 vol.%; on the other hand this is also a limitation that cannot produce higher CO_2_ concentrations using air as one of the gaseous sources.

### 3.2. Measurements of the Three O_2_ Sensors from Test-A

**Figure 6:**
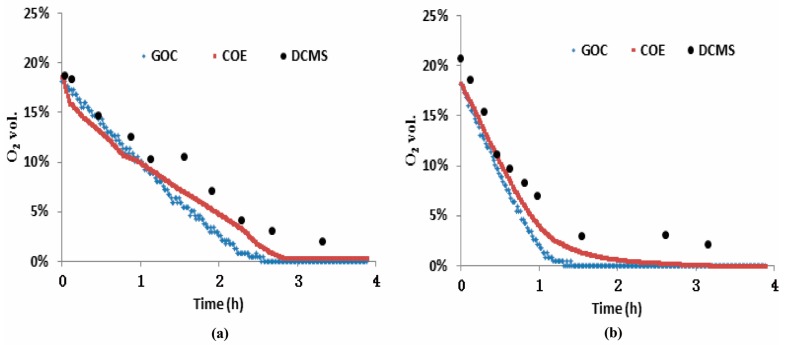
Measurements of the three types of O_2_ sensor came from Test-A, which mimic the ensiling phase related to O_2_ depletion process. (**a**) O_2_ variation at the low density packed (500 kg·m^−3^); and (**b**) that at the high density packed (800 kg·m^−3^).

**Figure 7:**
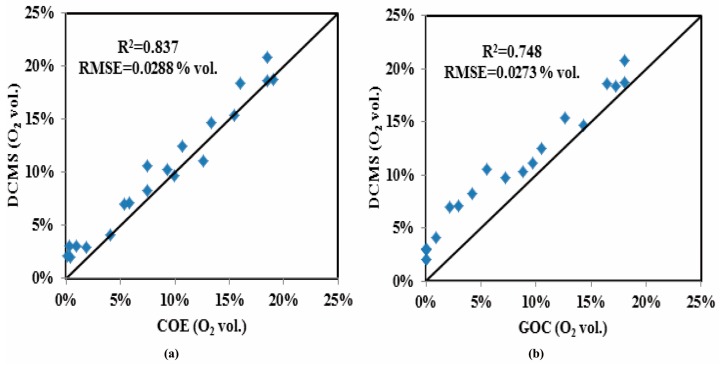
Measurement comparison between the DCMS *versus* the COE (**a**), and that between the DCMS *versus* the GOC (**b**) in Test-A.

Figure 6 contains two plots, one (Figure 6a) related to the low density packing and the other (Figure 6b) to the high density packing, each showing two traces of O_2_ depletion recorded by the COE and by the GOC from Test-A. Additionally, ten points in each plot came from the DCMS measurements. The measurements show that the O_2_ depletion process under the low BD (Figure 6a) is longer (about 2.5 h) than that (about 1.2 h) under the high BD (Figure 6b). This is because the low BD silage had more than double the porosity and therefore a commensurate supply of O_2_, with less than half the biomass or plant-cells carrying out respiration in comparison to the high BD barrel. The response time below 1 min using this type of COE has been verified in the literature [9]. As an additional result, the responsibility of the GOC used here is comparable to that of the COE. Over the period of Test-A, outputs of the COE and the GOC in each plot reported similar O_2_ variations, indicating that both sensors are capable of tracking the O_2_ dynamics during the ensiling phase. The spot-measurements of the DCMS also roughly followed the O_2_ depletion process. However, the DCMS measurements were overestimated relative to COE and GOC values in general. For assessing the overestimation of O_2_ from the DCMS in each barrel, first we rechecked the measurement accuracy of the DCMS with six additional measurements in air (mean value 20.7 vol.%) during the period of Test-A, and found its maximum absolute error ≤2 vol.% and its standard deviation (SD) was 5.4%. The latter, related to its measurement reproducibility, is less than the maximum SD error (±18%) in the manual [22]. The overestimation of the DCMS in the silage measurement was likely caused from the effect of high-level CO_2_, rather than from the chip analyzer. In addition, Figure 7a shows the data comparison with a 1:1 line between the DCMS and the COE (R^2^ = 0.837, RMSE = 0.0288 vol.%), and Figure 7b refers to that between the DCMS and the GOC (R^2^ = 0.748, RMSE = 0.0273 vol.%), respectively.

### 3.3. Measurements of the Three O_2_ Sensors from Test-B

**Figure 8:**
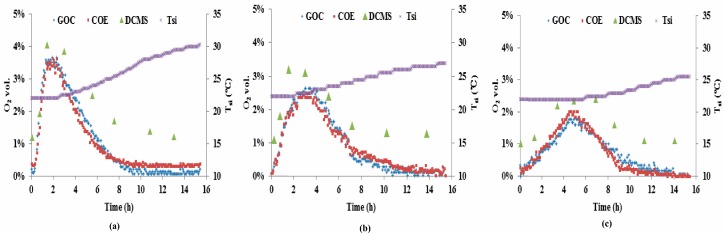
Measurements of the three types of O_2_ sensor came from the three barrels in Test-B, one barrel with an opened face (**a**) and the other two whose face was covered with 0.6 kg dry ice; (**b**) or 1.2 kg; (**c**). The silage temperature (T_si_) trace in each plot was recorded from the temperature sensor of the COE.

In terms of the experimental data from Test-B, Figure 8a refers to the barrel whose face remained open without dry ice covering the surface, whereas Figure 8b,c refer to the barrels whose faces were covered with the different amounts of dry ice. In general, all plots show similar trends with an O_2_ concentration rise-and-fall process. This can be interpreted from the literature [3,4], the O_2_ concentration increased rapidly 20-cm behind the face when the silo was opened because aerobic microorganisms in the silo were at relatively low levels. Once they multiplied to sufficiently high numbers near the surface, they consumed more of the O_2_ entering the silo, reaching maximum O_2_ concentration at 2, 3 and 5 h, respectively for the open and dry ice covered faces. The O_2_ concentration 20-cm behind the face returned to anaerobic conditions within 10 h in each barrel/case. In contrast, the peak of O_2_ in Figure 8a occurred earlier with larger amplitude than those in Figure 8b,c. This was likely due to the dry ice that played an impeding role to O_2_ penetrating the silage. When the dry ice was placed on the surface of the barrel, exposed to air at the room temperature, the dry ice began sublimating and releasing gaseous CO_2_, which is relatively heavier than O_2_. The outcome likely resulted in partial-pressure-based convective transport of CO_2_ away from the surface, opposing and thereby reducing the diffusive transport of O_2_ into the silage [5].

The silage temperature trace (T_si_) in each plot of Figure 8 showed an increased temperature response to the O_2_ rise-and-fall in each barrel. In the initial stage of Test-B, we did not capture a decrease of T_si_ in the zone of 20 cm behind the face in Figure 8b,c, suggesting that the effect of the dry ice on the temperature in deeper zone could be negligible. This is likely because the top layer of 20 cm had a high density and high water content, thereby resulting in much higher thermal capacity than air. Therefore, the thermal exchange for the dry ice mainly occurred with air above the boundary as it sublimated rapidly. In contrast, the high peak of O_2_ concentration (≈3.8 vol.%) in Figure 8a resulted in a relatively large increase in T_si_ (≈7.7 °C). In addition, there was an hour-long lag between the O_2_ diffusion peak and the T_si_ increase as shown in each plot. This is further evidence (i) that a rapid population increase in aerobic microorganisms consumed increasing O_2_ accompanying a significant heat release [3]; and (ii) that the measured O_2_ concentration is a more sensitive parameter for the detection of aerobic deterioration of biomass in silage production and management.

Similar to the DCMS measurements in Test-A, Figure 8 also reveals relative overestimation of O_2_ concentration (the DCMS measurements are denoted with solid Δ) in Test-B. According to the manual of the Dräger chip measurement system [22], its cross sensitivity is 0.5 vol.% ≤ CO_2_ at 1 vol.% of O_2_. Therefore, we inferred that the uncertainty of the DCMS measurements in each test partially stemmed from the influence of the CO_2_, resulting from the production of O_2_.

## 4. Conclusions

Based on the experimental results from the cross calibration of O_2_ *versus* CO_2_ and the two tests, three conclusions can be drawn:

(i) The cross calibration demonstrated that either the COE or the GOC has a linear relationship O_2_ concentrations and that they were insensitive to CO_2_ ranging from 0 to 15 vol.%;
(ii) Both the COE and the GOC provided continuous measurements of O_2_ concentration in maize silage. For the DCMS manual measurement, the general trends in O_2_ dynamics was captured when the measurement interval was sufficiently short;
(iii) In terms of measurement quality, the calibrated COE and the GOC sensors reported similarly high accuracies. The good-agreement of their measurements from Test-A and -B also confirm that the COE can be used not only for dissolved O_2_ in liquids, but also for gaseous O_2_ in maize silage. The reduced accuracy of the DCMS, especially at low O_2_ concentration, likely was caused by the relatively high levels of CO_2_, from the O_2_ production in the silage. In addition, the GOC economically seems the least costly option but it is unable to simultaneously measure O_2_ and T_si_, whereas the COE seems to have a high ratio of performance to cost. Although the DCMS is relatively expensive, the hand-held device readily and conveniently facilitates O_2_ concentration measurements at multiple points in varied silage treatments.
